# Speed Matters: Relationship between Speed of Eye Movements and Modification of Aversive Autobiographical Memories

**DOI:** 10.3389/fpsyt.2015.00045

**Published:** 2015-04-07

**Authors:** Suzanne Chantal van Veen, Kevin van Schie, Leoniek D. N. V. Wijngaards-de Meij, Marianne Littel, Iris M. Engelhard, Marcel A. van den Hout

**Affiliations:** ^1^Department of Clinical and Health Psychology, Utrecht University, Utrecht, Netherlands; ^2^Department of Methodology and Statistics of Social Science Research, Utrecht University, Utrecht, Netherlands

**Keywords:** EMDR, eye movements, autobiographical memory, working memory, vividness, emotionality

## Abstract

Eye movement desensitization and reprocessing (EMDR) is an efficacious treatment for post-traumatic stress disorder. In EMDR, patients recall a distressing memory and simultaneously make eye movements (EM). Both tasks are considered to require limited working memory (WM) resources. Because this leaves fewer resources available for memory retrieval, the memory should become less vivid and less emotional during future recall. In EMDR analogue studies, a standardized procedure has been used, in which participants receive the same dual task manipulation of 1 EM cycle per second (1 Hz). From a WM perspective, the WM taxation of the dual task might be titrated to the WM taxation of the memory image. We hypothesized that highly vivid images are more affected by high WM taxation and less vivid images are more affected by low WM taxation. In study 1, 34 participants performed a reaction time task, and rated image vividness, and difficulty of retrieving an image, during five speeds of EM and no EM. Both a high WM taxing frequency (fast EM; 1.2 Hz) and a low WM taxing frequency (slow EM; 0.8 Hz) were selected. In study 2, 72 participants recalled three highly vivid aversive autobiographical memory images (*n* = 36) or three less vivid images (*n* = 36) under each of three conditions: recall + fast EM, recall + slow EM, or recall only. Multi-level modeling revealed a consistent pattern for all outcome measures: recall + fast EM led to less emotional, less vivid and more difficult to retrieve images than recall + slow EM and recall only, and the effects of recall + slow EM felt consistently in between the effects of recall + fast EM and recall only, but only differed significantly from recall + fast EM. Crucially, image vividness did not interact with condition on the decrease of emotionality over time, which was inconsistent with the prediction. Implications for understanding the mechanisms of action in memory modification and directions for future research are discussed.

## Introduction

Trauma-exposed individuals may suffer from distressing and intrusive memories of their traumatic experience and some even develop post-traumatic stress disorder [PTSD; (1)]. Eye movement desensitization and reprocessing (EMDR) is a psychological treatment for PTSD, and its efficacy is comparable to cognitive behavioral therapy (2, 3). A key aspect of EMDR is that the patient makes bilateral eye movements (EM) during the retrieval of traumatic memory images. Empirical research has confirmed that this dual-task approach reduces the image vividness and emotional intensity of an aversive memory, both in healthy persons and in patients with PTSD [for a meta-analysis, see Ref. (4)]. Note that in EMDR analogue studies, a standard “dose” is typically used: EM with a speed of 1 cycle per second (1 Hz), in sets of 24 s [e.g., Ref. (5)]. This presumes that patients and aversive memories respond equally well to the same dual-task manipulation. Recent insights from experimental studies challenge the efficacy of this standardized procedure [e.g., Ref. (6–8)]. Therefore, the aim of the current research was to test whether titration based on image vividness enhances the effects of dual-task manipulation on aversive memories.

A range of experimental studies provides support for a working memory (WM) account to explain how EM decrease the image vividness and emotional intensity of negative memories [for an overview, see Ref. (8)]. More specifically, holding an emotional memory image in mind and performing EM will both tax the limited resources of WM (6, 9). Consequently, competition between these tasks should impair retrieval of the image with its accompanied details and emotions, and result in immediate decreased image vividness and emotional intensity of the memory before its return to long-term store. A laboratory model has been used to critically test this WM account. In this model, participants recall a negative memory image with or without simultaneously making EM. Image vividness and emotional intensity are measured before and after this intervention. Studies with healthy participants have shown that recall + EM decreases the vividness and/or emotionality of the recalled memory image, while recall without EM [recall only (RO)] does not (8). This effect has been replicated with other cognitively demanding tasks, such as counting backwards (10), attentional breathing (11), drawing a complex figure (6), and playing the computer game Tetris (12). Furthermore, it has not only been found for mental images of adverse past events, but also for mental images of imagined, aversive future events [e.g., Ref. (13)]. As predicted, tasks that barely tax WM, such as passively listening to sounds, are less effective than more cognitively demanding tasks [e.g., Ref. (14)]. These studies suggest that any dual-task that sufficiently taxes WM may decrease the vividness and/or emotionality of the recalled memory image.

Although many studies have shown that various dual-tasks affect emotional memory images, less is known about boundary conditions and optimization of the dual-task manipulation. The degree to which competition will occur between the WM load of the memory image and the WM load of the dual task partly depends on a person’s WM capacity. Individuals with a large WM capacity are expected to be relatively proficient in performing tasks simultaneously (multitasking). Because there will be less competition between the two tasks (memory image recall and dual task) for them, compared to individuals with a low WM capacity, the effects on memory image should be smaller. Evidence for a correlation between WM capacity and memory effects comes from a study by Gunter and Bodner (6) who found medium negative correlations between automated reading span scores – an indicator of WM span – and decreases of vividness and emotionality within the recall + EM condition. This finding was replicated by two other studies that showed that individual differences in WM span are negative related to beneficial effects of dual taxation of memory image recall + WM taxing: the larger the WM span, the smaller the benefits of recall + WM taxing (11, 15). To test the feasibility of the WM theory, Maxfield et al. (7) manipulated the speed of EM. As predicted, they found that fast EM (1.25 Hz) resulted in larger decreases in image vividness and emotional intensity than slow EM (1 Hz), and both EM conditions led to larger decreases than a control condition. The authors argue that fast EM are more difficult to perform (i.e., they are more taxing), which leads to larger effects on memory images. Although this is plausible, the actual WM load of the two speeds of EM was not measured. Also, the stimulus presentation was a repetition of short intervals of dual-task manipulation (left-right-left appearance of a stimulus). One could argue that this procedure tested the capability of task switching, rather than ongoing dual-task performance.

Contrary to the prediction that the higher the WM load of the dual task, the larger the dual-task manipulation effects, Gunter and Bodner (6) hypothesized that this relationship may not be linear. A task that is slightly taxing may not disrupt the memory image enough, and a task that is overly taxing might preclude holding the memory image in mind, thereby preventing competition effects. Therefore, they proposed an inverted U-shape function. In other words, too little or too much WM taxing may lead to smaller effects than WM taxing that is intermediate. This was tested and partially confirmed by Engelhard et al. (10), who found an inverted U-shape function for emotionality, but not for vividness. Participants recalled a negative memory image and performed one of four arithmetic tasks: exposure alone, or exposure with “simple” subtraction, “intermediate” subtraction, or “complex” subtraction. Prior to the memory experiment, the WM taxation of the four tasks was assessed using a discriminative reaction time (RT) task and the results indicated that the subtraction tasks indeed increasingly taxed the WM, with simple subtraction taxing WM the least and complex subtraction taxing the most. In line with the inverted U-shape hypothesis, emotional intensity of the memory image decreased more after recall during simple or intermediate subtraction than when after recall during complex subtraction or no subtraction. Results for vividness were in the expected direction, but were not significant. Variation was larger for vividness ratings than for emotionality ratings, and this latter may have caused the difference between the dependent variables. To sum up, research indicated that the WM load of that dual task is related to the effectiveness of the intervention, and that this relation presumably follows an inverted U-shape function. It is unclear, however, whether these effects are translated to various speeds of EM.

From a theoretical perspective, the effectiveness of the dual-task manipulation depends not only on the WM load of the dual task, but also on its interaction with the WM load of the memory. The WM load of the memory may be affected by variation in memory image vividness: highly vivid images are presumed to tax the WM more than less vivid images (16). Obviously, the degree of image vividness of aversive memories varies between individuals who have experienced the same situation and within one individual over time. These variations in image vividness may therefore influence the variation in WM load. According to the inverted U-shape hypothesis, if a memory image is highly vivid, a relative low degree of taxing WM by the dual task may produce insufficient blurring. Conversely, if the memory image is less vivid, strong WM taxing may preclude memory recall. Therefore, in order to maximize memory effects, the WM theory implies that there is a need for titration: highly vivid memories require a relatively high WM load and less vivid memories a lower load.

The current study used the WM framework to investigate the interaction between the WM load of the memory image and the WM load of the dual task. In study 1, we examined the WM load of five different speeds of EM. We hypothesized that faster EM are more taxing. This study resulted in the selection of two conditions: fast EM and slow EM. In study 2, participants recalled three highly vivid distressing memory images or three distressing memory images that were less vivid. These memories were randomized to each of three conditions: recall + fast EM, recall + slow EM, or RO. We predicted that (1) relative to RO, both EM conditions result in memory images that are less emotional, less vivid, and more difficult to retrieve, and more importantly (2) highly vivid memory images benefit more from fast than slow EM during recall, while less vivid memory images benefit more from slow than fast EM during recall.

## Study 1: WM Taxation of Different Speeds of EM

In order to select two speeds of EM that significantly differ in WM taxation, we tested the WM load of different speeds of EM in a within-subjects design. Participants performed a discrimination RT task during the performance of six tasks: five different speeds of EM and no EM. Slower RTs indicate the degree of taxation (17). In addition, participants were asked to hold six well-known images in mind (e.g., “your own kitchen”), while carrying out the same six tasks, and rated the vividness and difficulty to hold an image in mind during each task. We included vividness and difficulty ratings to test whether participants were still able to recall an image while simultaneously making the EM. We hypothesized that EM are more taxing than no EM, and that faster EM are more taxing than slower EM, resulting in larger RTs.

### Methods

#### Participants

Participants were recruited through advertisements at Utrecht University and the University of Applied Sciences (Hogeschool Utrecht), located at the same campus. Thirty-six participants (8 men, 28 women, *M*_age_ = 21.89, SD = 2.08) were tested, using no exclusion criteria. Two participants were removed from analyses due to technical problems. Participants received course credit or financial compensation for participation.

#### Materials and procedure

Participants were seated in front of a computer screen with a screen resolution of 1280 × 1024 at a distance of approximately 45 cm. OpenSesame 2.8.3 (18) was used to present stimuli. First, the low tone and high tone 1 s beeps (44.1 kHz) of the discrimination RT task were introduced. Beeps were administered to both ears through headphones using a constant volume. Participants pressed the *z*-key with their left index finger for low beeps and the /-key with their right index finger for high beeps. Beeps were presented randomly with a mean stimulus-onset asynchrony of 2.6 s (SD = 0.4). After a practice trial of 10 beeps, the experiment started. Participants were asked to categorize 20 low and 20 high beeps with or without making EM. In the EM conditions, a white 20 pixel dot appeared in the middle of a black screen and moved horizontally from side-to-side, with a movement amplitude of 461 pixel. The EM conditions had speeds of 0.4, 0.6, 0.8, 1.0, and 1.2 Hz (number of left-right-left cycles per second). Participants in the EM conditions were instructed to keep their head still and follow the dot with their eyes, and participants in the no EM were instructed to look at the middle of the screen (no dot was shown). The experimenter sat next to the participant and checked whether the EM were in accordance to the manipulation. If needed, the experimenter shortly repeated the instruction. In all conditions, the task was presented for a period of 106.6 s, adjusted to the average total time of beeps plus one (41 s × 2.6 s). The order of the speed of EM was randomly assigned, but each participant completed all six conditions.

To test whether participants were still able to recall a mental image while they simultaneously made EM, participants received the same condition again immediately after the RT trial, but instead of responding to beeps, they were instructed to simultaneously hold a well-known image in mind as vividly as possible. After 24 s, participants rated the vividness and difficulty of that image during manipulation on a visual analog scale (VAS), ranging from 0 (*not vivid/difficult at all*) to 100 (*very vivid/difficult*). The well-known mental images were the participant’s kitchen, bathroom, bed, wardrobe, front door, and bicycle. Latin-square counterbalancing was used to order the sequence of these six images.

#### Design and data analyses

To test the WM taxation of the various speeds of EM, relative to no EM, a repeated measure analysis of variance (ANOVA) was performed with speed of EM as within-subjects factor and average RT as outcome measure. The first and last beeps were excluded from the calculation of the average RT, to exclude potential transition delays. Differences in vividness and difficulty of holding an image in mind between conditions were analyzed by two repeated measures ANOVAs with speed of EM as within-subjects factor and vividness or difficulty as outcome measure. Alpha levels of 0.05 were used; they were one-tailed for tests crucial to the hypothesis. For small violations of sphericity, the degrees of freedom of the *F*-distribution were corrected with either Green–Geisser (0.70 ≥ ε < 0.75) or Huynh–Feldt corrections (ε ≥ 0.75). More severe violations (0.70 < ε) were corrected using a multivariate test statistic (Pillai-Bartlett trace; *V*).

### Results

**Figure 1:**
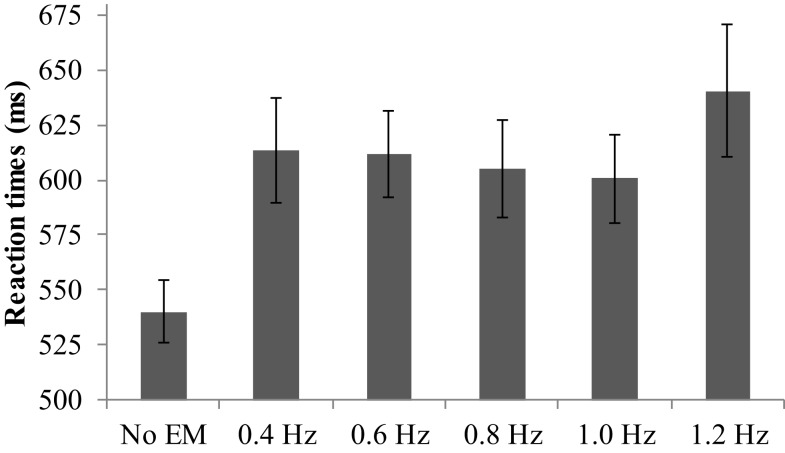
**Mean reaction times (ms) and SEs for the different speeds of EM and no EM**.

The average RT varied significantly across conditions, *V* = 0.48*, F*(5, 29) = 5.40, *p* = 0.001, $\eta_{p}^{2}=0.48$ (see Figure 1). Pairwise comparisons showed that all EM conditions during the RTT yielded increased RTs compared to no EM, range *M*_dif_ = 60–100, *p*s < 0.001. Furthermore, simple contrasts indicated that RTs in the fastest condition (1.2 Hz) were significantly greater than in the 0.8 Hz EM condition, *F*(1, 33) = 4.50, *p* = 0.02, $\eta_{p}^{2}=0.12$, or 1.0 Hz EM condition, *F*(1, 33) = 4.22, *p* = 0.03, $\eta_{p}^{2}=0.11$. The average number of correct items was high (*M*_range_ = 36–37 out of 39) and did not differ between the conditions.

**Figure 2:**
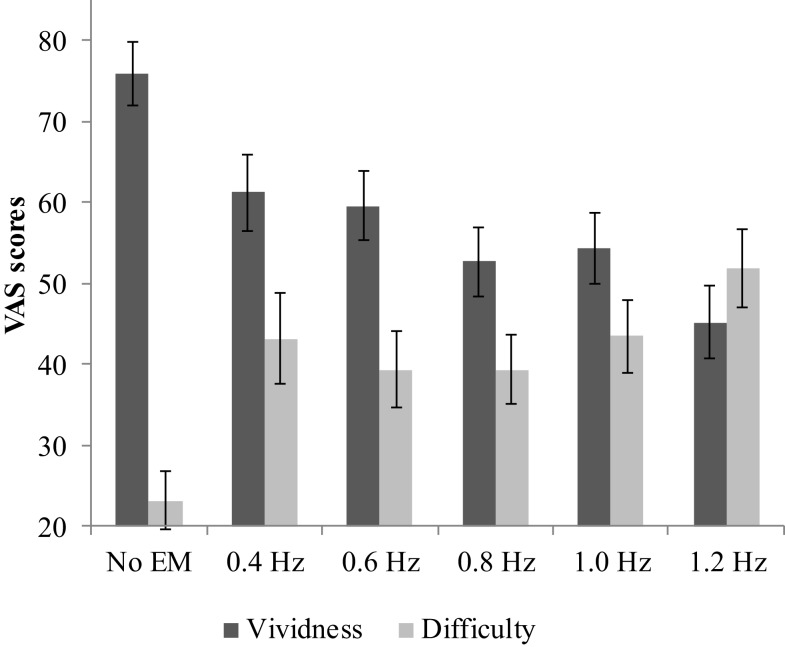
**Mean VAS scores and SEs for vividness and difficulty for the different speeds of EM and no EM**.

Average vividness scores differed between conditions, *F*(5, 29) = 11.42, *p* < 0.001, $\eta_{p}^{2}=0.26$ (see Figure 2). Pairwise comparisons showed that all EM conditions decreased the vividness of the image compared with no EM, *M*_dif_ = 15–31, *p*s < 0.002. The relation between WM taxation and vividness indicated a clear negative linear relationship: vividness decreased as WM taxation increased. Difficulty retrieving the image while performing the dual-task differed between the conditions, *F*(3.58, 118.15) = 9.25, *p* < 0.001, $\eta_{p}^{2}=0.22$ (see Figure 2). Pairwise comparisons showed that it increased for all EM conditions compared with no EM, *M*_dif_ = 16–29, *p*s < 0.002. Simple contrasts indicated that for both vividness and difficulty ratings, the 1.2 Hz EM condition differed significantly from the 0.8 and 1.0 Hz condition, *p*s < 0.05. The highest speed (1.2 Hz) resulted in a mean vividness of 45.20 (SD = 26.46) and mean difficulty of 51.90 (SD = 27.91).

### Discussion study 1 and introduction study 2

In line with previous research [e.g., Ref. (11)], all EM conditions resulted in slower RTs compared to no EM, indicating that performing EM indeed taxes WM. Between the EM conditions, EM of 1.2 Hz produced more WM taxation, lower image vividness, and higher difficulty to retrieve the image during manipulation compared to EM of 0.8 and 1.0 Hz. The 0.8 and 1.0 Hz conditions did not differ from each other on any of the outcome measurements. Since 1.0 Hz is the standard EM speed in EMDR, this could be considered an “intermediate” speed of EM. To keep the amount of variation equal on both ends, we selected 0.8 Hz for the slow EM condition and 1.2 Hz for the fast EM condition. Study 2 tested whether the WM load of EM interacts with the image vividness of a negative memory.

## Study 2: Interaction between Speed of EM and Image Vividness

### Methods

#### Participants

We recruited 92 undergraduate students through advertisements at the Utrecht University and the University of Applied Sciences (Hogeschool Utrecht). Exclusion criteria were knowledge about EMDR, prior participation in an experiment from our laboratory that required participants to recall memories, or medication use that may affect concentration, such as benzodiazepines. We excluded 20 students based on these exclusion criteria. The final sample consisted of 72 participants (22 male, 50 female, *M*_age_ = 22.40, SD = 3.81). They were randomly assigned to one of two groups: “highly vivid memories,” *n* = 36; “less vivid memories,” *n* = 36. Participants received course credit or financial compensation.

#### Materials and general procedure

Participants were tested individually in a quiet room. After providing written informed consent, participants were interviewed by the experimenter (see below). Participants selected three negative memories following the procedure used by van den Hout et al. (5). Next, in line with the Dutch EMDR standard protocol (19), they selected a target image of each memory. During the second half of the experiment, participants were seated behind a computer with a screen resolution of 1280 × 1024 at a distance of about 45 cm. OpenSesame 2.8.3 (18) was used to present stimuli.

#### Memory selection

During the first half of the experiment, participants selected three negative memories that were at least 1 week old and still evoked relevant feelings (i.e., fear/anxiety/sadness). Participants in the highly vivid memories group were instructed to select three negative “memories that are very clear and detailed,” and participants in the less vivid memories group were instructed to select three negative “memories that are relatively vague and low on details.” If participants found it difficult to select memories, the experimenter presented a list of examples (e.g., eye-witness of a traffic-accident, a job rejection, an argument with a family member), and stressed that vividness of memories is subjective, so the example memories were merely given to stimulate the selecting process. Participants wrote down the content of each memory on a card and indicated the vividness (with 0 *not at all vivid* to 100 *very vivid*) and emotionality (0 *not at all unpleasant* to 100 *very unpleasant*) of each memory. The experimenter checked if these ratings were within the intended range, which was 70–100 for vividness in the highly vivid memories group, 30–60 for vividness in the less vivid memories group, and 50–90 for emotionality in both groups. If it was not, the experimenter asked the participants to select another memory. Memories were ranked based on vividness ratings (1 = *most vivid*, 3 = *least vivid*, 2 = *in between*). The order of the target image selection, as well as the order of the conditions, was counterbalanced based on this ranking.

#### Target image selection

Next, the experimenter asked the participants to describe the memory in global story lines. Then, the experimenter asked the participant to identify the worst moment of this memory and describe this moment as a still image (i.e., “target image”). The participants assigned a descriptive, relatively neutral label to each target image, to act as a cue during the experiment.

#### Experiment

Then, the participants performed a pre-test, an intervention phase, and a post-test for each condition. In the pre-test, participants recalled their target image for 10 s and gave ratings of emotional valence, vividness, and difficulty of retrieving the target image on the VAS (ranging from 0 *not at all unpleasant/vivid/difficult* to 100 *very unpleasant/vivid/difficult*). In the intervention phase, they recalled their target image six times for 24 s, with 10 s rest periods in between. Each rest period ended with a 2 s instruction to recall the target image again. In each EM condition, participants held their head still and looked at a horizontally moving white dot (20 pixel) on a black screen. The dot had a movement amplitude of 461 pixel, and a speed of 0.8 Hz in the slow EM condition and 1.2 Hz in the fast EM condition. In the RO condition, participants recalled the target image and looked at the black screen. If participants moved their head or eyes incorrectly, the experimenter briefly repeated the instructions. The post-test was immediately after the intervention. In the post-test, participants again brought the target image to mind for a 10 s period and rated the same VAS.

### Results

#### Manipulation check

During memory selection, all participants managed to select three memories that matched the vividness criteria. However, a manipulation check based on the vividness ratings in the pre-test indicated that only 33 participants (45.8%) had three target images within the vividness range of their condition. For the less vivid memories group, vividness scores during the memory selection were significantly lower (*M* = 50.31, SD = 6.07) compared to the pre-test ratings of the target image [*M* = 63.64, SD = 13.05; *t*(35) = -5.82, *p* < 0.001]. For the highly vivid memories group, vividness scores during memory selection and the pre-test did not differ from each other (*M*_selection_ = 79.85, SD = 5.24; *M*_pre-test_ = 79.67, SD = 8.13, *p* = 0.91). Because our manipulation check indicated that target image vividness did not match the intended group criteria (highly vivid memories vs. less vivid memories), we analyzed the data on the memory level instead of on the participant (group) level.

#### Analysis strategy

**Figure 3:**
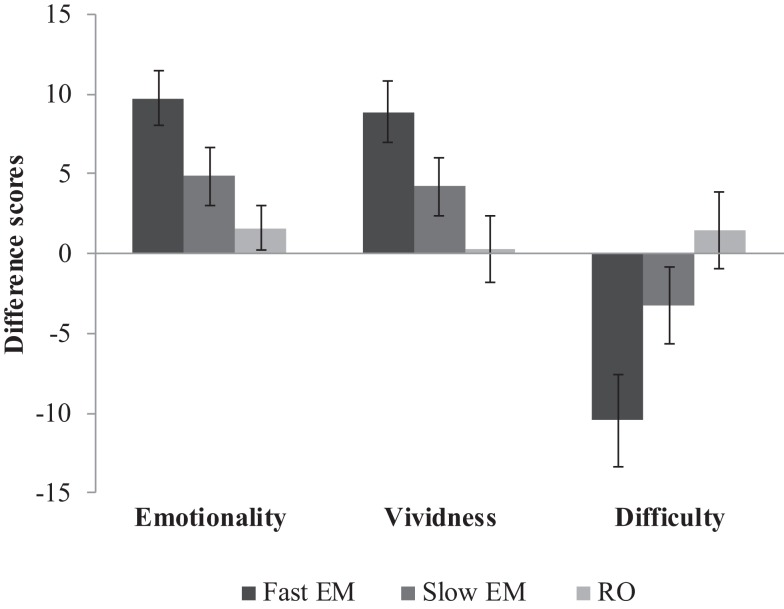
**Mean difference scores (post-test minus pre-test) and SEs of fast EM, slow EM, and RO on emotionality, vividness, and difficulty**.

Memories were nested within participants. Therefore, we analyzed the data with multilevel modeling using three levels: 432 repeated measures (level 1) of 216 memories (level 2), nested within 72 participants (level 3). We conducted the analyses with Hierarchical Linear and Non-linear Modeling, version 6 [HLM6, Ref. (20)]. For our first hypothesis that EM would decrease emotionality and vividness, and increase the difficulty of retrieving the memory image more than RO, we analyzed emotionality, vividness, and difficulty over time between the conditions. Figure 3 shows the mean difference scores (post-test minus pre-test) and SEs of all three conditions on emotionality, vividness, and difficulty. Table 1 shows the fixed and random parts of the same multilevel model applied to each outcome measure. Condition was coded as dummy variable, with RO as reference condition. Therefore, the variable *RO_slowEM* indicated the difference between RO and the slow EM condition, and *RO_fastEM* indicated the difference between RO and the fast EM condition. The mixed equation for each model was: outcome variable_ijk_ = β_00_ + β_10_ (*time*)_ijk_ + β_01_ (*RO_slowEM*)_jk_ + β_02_ (*RO_fastEM*)_jk,_ + β_11_ [(*RO_slowEM*)_ijk_ × (*time*)_ijk_] + β_12_ [(*RO_fastEM*)_ijk_ × (*time*)_ijk_] + *v*_0k_ + *u*_0jk_ + *u*_1jk_ (*i* = time, *j* = memory, *k* = person).

**Table 1:** **Fixed and random parts of Model 1 (emotionality over time between conditions), Model 2 (vividness over time between conditions), and Model 3 (difficulty over time between conditions)**.

		Model 1		Model 2		Model 3	
		Emotionality		Vividness		Difficulty	
		β	SE	β	SE	β	SE
**FIXED PART**							
Intercept	β_00_	72.59*	1.54	72.69*	1.97	40.17*	2.88
*RO_slowEM*	β_01_	− 2.31	1.87	− 1.94	2.18	0.28	3.02
*RO_fastEM*	β_02_	1.46	1.87	− 1.17	2.18	− 2.47	3.02
Time	β_10_	− 1.57	1.63	− 0.28	1.92	− 1.57	2.54
*RO_slowEM*	β_11_	− 3.28	2.32	− 3.91	2.72	4.83	3.60
*RO_fastEM*	β_12_	− 8.16*	2.32	− 8.63*	2.72	12.04*	3.60
**RANDOM PART**							
$\sigma_{v0k}^{2}$		45.99*		107.67*		267.98*	
$\sigma_{u0\mathrm{jk}}^{2}$		125.74*		171.10*		328.41*	
$\sigma_{u1\mathrm{jk}}^{2}$		193.09*		265.42*		467.83*	
Deviance		3457.23		3567.08		3819.20	

*In all models, RO was the reference condition*.

*p < 0.05.

The second hypothesis was that highly vivid memory images benefit more from fast EM than slow EM during recall, and less vivid memory images benefit more from slow EM than fast EM during recall. To test the difference between slow and fast EM, we used slow EM as reference condition. Accordingly, the dummy *slowEM_RO* indicated the difference between slow EM and RO, and the dummy *slowEM_fastEM* indicated the difference between slow EM and fast EM. To observe the three-way interaction between pre-test vividness, condition and time, interaction variables between the centered *pre-test vividness* variable and the dummy condition variables were added as predictor for the intercept at the second level and as predictor for the slope of time at the first level (Model 4). Support for the hypothesis should materialize as a significant negative coefficient in predicting the slope of time for the variable *pre-test vividness* × *slowEM_fastEM*: the higher the vividness of the target image at pre-test, the more decrease in emotionality for the fast EM condition compared to the slow EM condition. Likewise, the lower the vividness of the target image at pre-test, the less decrease in emotionality for the fast EM condition when compared to the slow EM condition.

The mean pre-score vividness was 71.66 with a pile-up of scores on the right of the distribution (range 30.75–98.63, SD = 16.82, *N* = 216, *z*_skewness_ = −3.68*, z*_kurtosis_ = −1.39). To establish that there was no detrimental effect of the skewed distribution on the analyses, the distribution of errors of the second and third level were inspected for the final models. No abnormalities were detected.

#### Emotionality over time between conditions

Memories in the RO condition were stable in emotionality over time, β_10_ = −1.57, *p* = 0.340. Contrary to expectations, memories in slow EM did not decrease emotionality when compared to RO, β_11_ = −3.28, *p* = 0.158. However, fast EM did result in a larger decrease of emotionality over time than RO, β_12_ = −8.16; *t*(213) = −3.52, *p* = 0.001: post-test scores were lower (predicted mean = 65.89) than pre-test scores (predicted mean = 74.04). Next, to test whether the fast EM condition differed from the slow EM condition, we analyzed the same model with slow EM as reference condition. This revealed that fast EM led to larger decreases in emotionality than did slow EM, β_12_ = −4.87; *t*(213) = −2.10, *p* = 0.04. This means that fast EM were superior to both RO and slow EM in decreasing the emotional intensity of memory images. Finally, Table 1 (Model 1) summarizes the random components of the model. Emotionality ratings of the memories varied significantly across participants $\left(\sigma_{v0k}^{2}\right)$, across memories within participants $\left(\sigma_{u0\mathrm{jk}}^{2}\right)$, and across time within memories within participants $\left(\sigma_{u1\mathrm{jk}}^{2}\right)$, *p*s < 0.001.

#### Vividness over time between conditions

Similar to the differences between conditions on emotionality, memories in RO showed stable vividness ratings over time, β_10_ = −0.28, *p* = 0.885, memories in slow EM did not decrease vividness compared to RO, β_11_ = −3.91, *p* = 0.151, while memories in fast EM yielded a significant difference compared to RO, β_12_ = −8.63; *t*(213) = −3.18, *p* = 0.002: post-test scores were lower (predicted mean = 62.89) than pre-test scores (predicted mean = 71.52; Model 2, Table 1). A re-run of the model with slow EM as reference condition revealed that fast EM showed a non-significant trend toward larger decreases in vividness ratings, β_12_ = −4.72; *t*(213) = −1.74, *p* = 0.083. So, it seems that memory images that were recalled while making fast EM decreased more in vividness than images that were recalled while making slow EM or were only recalled without dual task. Vividness ratings of the memories varied significantly across participants $\left(\sigma_{v0k}^{2}\right)$, across memories within participants $\left(\sigma_{u0\mathrm{jk}}^{2}\right)$, and across time within memories within participants $\left(\sigma_{u1\mathrm{jk}}^{2}\right)$, *p*s < 0.001.

#### Difficulty over time between conditions

Likewise, the same pattern between the conditions was found for the difficulty of retrieving the target image. Memories in RO showed a stable score over time, β_10_ = −1.57, *p* = 0.539, slow EM did not increase difficulty more than RO, β_11_ = 4.83, *p* = 0.182, but fast EM did increase difficulty recalling the memory compared to RO, β_12_ = 12.04; *t*(213) = 3.34, *p* = 0.001: post-test scores were higher (predicted mean = 49.74) than pre-test scores (predicted mean = 37.70; Model 3, Table 1). A re-run of the model with slow EM as reference condition revealed that fast EM led to larger increases in difficulty than did slow EM, β_12_ = −4.83; *t*(213) = 2.00, *p* = 0.046. So, fast EM caused more difficulty in retrieving the memory image after intervention than both RO and slow EM. Again, difficulty ratings varied significantly across participants $\left(\sigma_{v0k}^{2}\right)$, across memories within participants $\left(\sigma_{u0\mathrm{jk}}^{2}\right)$, and across time within memories within participants $\left(\sigma_{u1\mathrm{jk}}^{2}\right)$, *p*s < 0.001.

#### Interaction between pre-test vividness, condition, and emotionality over time

Model 4 revealed that the coefficient of *pre-test vividness* × *slowEM_fastEM* in predicting the slope for time was −0.14 (SE = 0.14) and not significant, *t*(210) = −1.05, *p* = 0.297. So, inconsistent with our predictions, pre-test vividness did not interact with condition on changes in emotionality ratings.

## Discussion

This study aimed to examine whether WM load of a dual-task carried out during memory image recall interacts with WM load of that memory on reducing its emotional intensity. We found a consistent pattern for all outcome measures: high WM taxation (recall + fast EM) was superior to low WM taxation (recall + slow EM) and no WM taxation (recall only; RO), and the effects of low taxation felt consistently in between the effects of high taxation and RO, but only differed significantly from high taxation. High WM taxation during recall produced memory images that were less vivid, less emotional, and were more difficult to retrieve after the intervention. This is in line with WM theory: the more taxing a dual-task is, the more a memory image degrades. Crucially, image vividness did not interact with condition (high taxation vs. low taxation) with regard to the decrease of emotionality over time. Thus highly vivid and less vivid images showed the same responsiveness to dual-task manipulation: both images benefited the most from high WM taxation during recall.

The finding that recall + dual WM taxing reduced memory image vividness and emotionality, compared to RO, is in line with a large body of experiments [see Ref. (8), for an overview]. More specifically, this study replicated the findings of Maxfield et al. [Ref. (7); experiment 2], who also found that fast EM (1.25 Hz) yielded stronger reductions in memory image vividness and emotional intensity than slow EM (1.0 Hz) and no EM. They contributed this difference in effects to presumed variation in WM taxation, but did not experimentally assess the WM taxation of both EM tasks. We extended their design and used RT methods to select two speeds of EM that significantly differed in WM taxation (study 1). We found the same superiority effects of fast EM compared to slow EM on image vividness and emotional intensity. Furthermore, we measured the difficulty of retrieving the memory image before and after the intervention and found that the higher the WM taxation, the more difficult it was to retrieve the memory image after intervention. Together these studies provide strong evidence for the WM theory in explaining the effectiveness of dual-task manipulation on memory modification. Low WM taxation produced memory effects in the same direction as high WM taxation; however, only high WM taxation was effective enough to produce memory effects that differed significantly from a control condition after a short intervention (6 × 24 s).

The superiority of the 1.25 Hz condition over the 1.0 Hz condition in the study of Maxfield et al. (7) suggests a linear relationship: higher WM taxing results in larger memory effects than lower WM taxing. However, according to the inverted U-curve hypothesis (6), strongest effects are found when competition between memory recall and the dual-task use approximately the same amount of WM resources. Too little taxation of the dual task will leave too many resources available for vivid memory recall and its accompanying emotions, while too much taxation of the dual task prevents the memory from being recalled. In a recent study, an inverted U-curve pattern was observed for emotionality, but not for vividness (13). In the current study, we examined whether the EM intervention would be more effective if the load of the dual task is matched with the load of the memory. We hypothesized that highly vivid memory images would benefit more from fast EM than from slow EM, and less vivid memory images would benefit more from slow EM than from fast EM. Contrary to these hypotheses, there were no interactions between image vividness and dual task WM taxation. Several explanations will be discussed.

First, it could be argued that slow EM were not sufficiently demanding and did not trigger the hypothetical threshold of the inverted U-curve. However, results of study 1 showed that slow EM tax WM more than no EM. Furthermore, EM with a speed of 0.8 Hz had comparable WM taxation as EM with a speed of 1.0 Hz. Because of these results, and because many laboratory studies have found memory effects with 1.0 Hz, which can be considered the “standard speed” (4), the argument that slow EM were not taxing enough seems not plausible. The fact that in study 2 slow EM was attended by effects on memory that were in the same direction as fast EM could indicate a dosage effect: the more cognitive demanding a dual task, the larger the memory effects. It could be hypothesized that an extended duration (e.g., more sets of recall + EM) would lead to a difference between slow EM and RO. For example, Leer et al. (21) found that eight sets of recall with EM, compared to RO, caused a decrease in emotionality at a 24 h follow-up test, while four sets did not.

There may be a second explanation for the absence of an interaction effect between dual task load condition and image vividness. Possibly, image vividness does not influence the amount of WM load. In the present study, WM load of the memory image itself was not measured. However, the relation between WM and vividness of imagery was examined in series of experiments with dual task manipulations by Baddeley and Andrade (16). It was concluded that vividness of imagery reflects the richness of representation in WM. Moreover, more recent evidence indicates that emotional memories tax WM to a greater extent than neutral memories [Ref. (22); see Discussion]. Based on these previous studies, it seems justified to presume that image vividness affects the degree of WM taxation. In order to fully clarify this issue, it would be interesting to have participants recall images with a wide variation of vividness while performing a simple RT task. This would enable us to measure the cognitive demanding qualities of the memory image.

Alternatively, because WM load of the dual task did not interact with WM load of the memory image, one may question whether individuals are actually able to hold a memory image in mind while performing a dual task. The WM account is derived from the WM theory by Baddeley and Hitch (23) in which three memory components are described: an attentional control system (central executive) and two slave storage systems (visuospatial sketch path and phonological loop). Later, Baddeley and Andrade (16) added a fourth component: the episodic buffer, which is a limited-capacity temporary storage system that allows integration from both the slave systems with material from long-term memory. The central executive is thought to control the retrieval and modification of information that is temporally stored in the episodic buffer. The central executive may therefore influence the content of information, by directing attention to a specific source: the slave systems or long-term memory. Based on this model, it seems likely that during a dual-task manipulation, the central executive is involved in attending to both tasks, while the temporal storage and integration of information takes place in the episodic buffer. During dual-task manipulation in our study, information is retrieved from long-term memory and maintains active in the episodic buffer. This process of constant reactivation to maintain an image active requires much effort [see Ref. (24)]. A crucial question is whether performing EM *interferes* with the memory material due to integration of both tasks in the episodic buffer or whether division of attention between the two tasks by the central executive *inhibits* the memory material to be fully activated. If the former is true, then maximizing the complexity of a cognitive demanding task may leave almost no resources available for active recall of material from long-term memory and therefore there will be little interference. If the latter is true, then maximizing the complexity of a cognitive demanding task may lead to memory retrieval strategies, such as rapid shifting between tasks, which could lead to partial exposure to the memory and result in devaluation of the memory. More fundamental studies are needed to investigate these hypotheses about the cognitive processes that underlie the effects of dual task procedures on emotional memories.

Finally, there were some short-comings of the current study. First, we selected memories high or low in vividness, but the image vividness changed during the experiment prior to the intervention. This may have resulted in unreliable conditions. That is, selecting the target image seemed to inflate its vividness. We therefore analyzed the data on the memory level instead of on the person level and used multilevel modeling to correct for the assumption violation of independent data. A strength of multilevel modeling is that it allowed the use of vividness as a continuous predictor, and therefore provides more detailed information than a dichotomous division in target image vividness. Second, the general ability to use mental imagery was not measured. Individual differences in imagery may influence the effectivity of dual task manipulation. Future studies could test this influence through assessment of the ability to use mental imagery with the Spontaneous Use of Imagery Scale [SUIS; Ref. (25)] or, more specified to visual imagery, the revised version of the Vividness of Visual Imagery Questionnaire [VVIQ-2; Ref. (26)]. Third, vividness and emotionality ratings were based on subjective ratings. Psychophysiological measures could be used as objective indicator of memory emotionality [e.g., Ref. (12, 27, Kearns & Engelhard, Submitted)]. Fourth, the current study did not use standardized compliance measures: we manipulated the speed of the dot moving from left to right and corrected the participant if they did not follow the dot properly, but we did not test the actual speed of participants’ EM. Using electro-oculogram analysis might help here. Finally, we only analyzed the immediate influence of dual-task manipulation on memory modification. Research has yet to determine whether memory modification effects are maintained over time (21).

In sum, we found consistent effect patterns that are in line with WM theory: the more cognitively demanding the dual task, the more an aversive memory image can be modified, in that these images become less emotional, less vivid, and more difficult to retrieve. In our study, WM load of the memory – operationalized by image vividness – did not interact with the WM load of the dual task. Therefore, we found no evidence for the inverted U-curve hypothesis proposed by Gunter and Bodner (6). Further research is needed to critically test whether the inverted U-curve hypothesis does occur for other intra-individual variables, such as differences in WM capacity. Unraveling the complexities of WM theory may provide a better idea of how titration between the recalled memory image and the WM load of the dual task may be optimized.

## Conflict of Interest Statement

The authors declare that the research was conducted in the absence of any commercial or financial relationships that could be construed as a potential conflict of interest.

## Abbreviations

EM, eye movements; EMDR, eye movement desensitization and reprocessing; RO, recall only; WM, working memory.

## References

[1] Kessler RC Tat Chiu W Demler O Walters EE Prevalence, severity, and comorbidity of 12-month DSM-IV disorders in the national comorbidity survey replication. Arch Gen Psychiatry (2005) 62:617–27 10.1001/archpsyc.62.6.617 15939839 PMC2847357

[2] Bisson JI Ehlers A Matthews R Pilling S Richards D Turner S. Psychological treatments for chronic post-traumatic stress disorder systematic review and meta-analysis. Br J Psychiatry (2007) 190(2):97–104. 10.1192/bjp.bp.106.021402 17267924

[3] Seidler GH Wagner FE. Comparing the efficacy of EMDR and trauma-focused cognitive-behavioral therapy in the treatment of PTSD: a meta-analytic study. Psychol Med (2006) 36(11):1515–22. 10.1017/S0033291706007963 16740177

[4] Lee CW Cuijpers P. A meta-analysis of the contribution of eye movements in processing emotional memories. J Behav Ther Exp Psychiatry (2013) 44(2):231–9. 10.1016/j.jbtep.2012.11.001 23266601

[5] van den Hout M Muris P Salemink E Kindt M. Autobiographical memories become less vivid and emotional after eye movements. Br J Clin Psychol (2001) 40(2):121–30. 10.1348/014466501163571 11446234

[6] Gunter RW Bodner GE. How eye movements affect unpleasant memories: support for a working-memory account. Behav Res Ther (2008) 46(8):913–31. 10.1016/j.brat.2008.04.006 18565493

[7] Maxfield L Melnyk WT Hayman GC. A working memory explanation for the effects of eye movements in EMDR. J EMDR Pract Res (2008) 2(4):247–61. 10.1016/j.jbtep.2013.07.002 23892070

[8] van den Hout MA Engelhard IM How does EMDR work? J Exp Psychopathol (2012) 3(5):724–38 10.5127/jep.028212

[9] Andrade J Kavanagh D Baddeley A. Eye-movements and visual imagery: a working memory approach to the treatment of post-traumatic stress disorder. Br J Clin Psychol (1997) 36(2):209–23. 10.1111/j.2044-8260.1997.tb01408.x 9167862

[10] Engelhard IM van den Hout MA Smeets MA. Taxing working memory reduces vividness and emotional intensity of images about the Queen’s day tragedy. J Behav Ther Exp Psychiatry (2011) 42(1):32–7. 10.1016/j.jbtep.2010.09.004 21074004

[11] van den Hout MA Engelhard IM Beetsma D Slofstra C Hornsveld H Houtveen J EMDR and mindfulness: eye movements and attentional breathing tax working memory and reduce vividness and emotionality of aversive ideation. J Behav Ther Exp Psychiatry (2011) 42:423–31. 10.1016/j.jbtep.2011.03.004 21570931

[12] Engelhard IM van Uijen SL van den Hout MA. The impact of taxing working memory on negative and positive memories. Eur J Psychotraumatol (2010) 1-8:5623. 10.3402/ejpt.v1i0.5623 22893797 PMC3402003

[13] Engelhard IM van den Hout MA Dek ECP Giele CL van der Wielen JW Reijnen M Reducing vividness and emotional intensity of recurrent “flashforwards” by taxing working memory: an analogue study. J Anxiety Disord (2011) 25:599–603. 10.1016/j.janxdis.2011.01.009 21376527

[14] van den Hout MA Rijkeboer MT Engelhard IM Klugkist I Hornsveld H Toffolo M Tones inferior to eye movements in the EMDR treatment of PTSD. Behav Res Ther (2012) 50:275–9. 10.1016/j.brat.2012.02.001 22440458

[15] van den Hout MA Engelhard IM Smeets MAM Hornsveld H Hoogeveen E de Heer E Counting during recall: taxing of working memory and reduced vividness and emotionality of negative memories. Appl Cogn Psychol (2010) 24(3):303–11. 10.1016/j.jbtep.2011.03.004 21570931

[16] Baddeley AD Andrade J Working memory and the vividness of imagery. J Exp Psychol (2000) 129(1):126–45 10.1037/0096-3445.129.1.126 10756490

[17] Bower GH Clapper JP Experimental methods in cognitive science. In: Posner MI, editor. Foundations of Cognitive Science. Cambridge: MIT Press (1989). p. 245–300.

[18] Mathôt S Schreij D Theeuwes J. OpenSesame: an open-source, graphical experiment builder for the social sciences. Behav Res Methods (2012) 44(2):314–24. 10.3758/s13428-011-0168-7 22083660 PMC3356517

[19] de Jongh A ten Broeke E Handboek EMDR: Een Geprotocolleerde Behandelmethode voor de Gevolgen van Psychotrauma [EMDR Handbook: A Protocol Treatment for the Effects of Psychological Trauma]. Amsterdam: Pearson (2012).

[20] Raudenbush SW Bryk AS Congdon R HLM 6 for Windows [Computer software]. Skokie, IL: Scientific Software International, Inc (2004).

[21] Leer A Engelhard IM van den Hout MA. How eye movements in EMDR work: changes in memory vividness and emotionality. J Behav Ther Exp Psychiatry (2014) 45(3):396–401. 10.1016/j.jbtep.2014.04.004 24814304

[22] van den Hout MA Eidhof MB Verboom J Littel M Engelhard IM. Blurring of emotional and non-emotional memories by taxing working memory during recall. Cogn Emot (2014) 28(4):717–27. 10.1080/02699931.2013.848785 24199660

[23] Baddeley AD Hitch G Working memory. Psychol Learn Motiv (1974) 8:47–89.

[24] Smeets MA Dijs MW Pervan I Engelhard IM Van den Hout MA. Time-course of eye movement-related decrease in vividness and emotionality of unpleasant autobiographical memories. Memory (2012) 20(4):346–57. 10.1080/09658211.2012.665462 22537073

[25] Reisberg D Pearson DG Kosslyn SM Intuitions and introspections about imagery: the role of imagery experience in shaping an investigator’s theoretical views. Appl Cogn Psychol (2003) 17(2):147–60 10.1002/acp.858

[26] Marks DF New directions for mental imagery research. J Ment Imagery (1995) 19:153–67.

[27] Barrowcliff AL Gray NS Freeman TC MacCulloch MJ Eye-movements reduce the vividness, emotional valence and electrodermal arousal associated with negative autobiographical memories. J Forens Psychiatry Psychol (2004) 15(2):325–45 10.1080/14789940410001673042

